# In vitro and molecular docking and analysis of isoxazoline derivatives with DPPH 

**DOI:** 10.6026/97320630016807

**Published:** 2020-11-30

**Authors:** C Geetha, PR Rajakumar

**Affiliations:** 1 PG & Research Department of Chemistry, Government Arts College, C. Mutlur 608 102, Chidambaram, India

## Abstract

A series of isoxazoline derivatives (4a-i) was synthesized from the reaction of 3-(4-fluorophenyl)-1-phenylprop-2-en-1-one derivatives (4a-i) and hydroxylamine hydrochloride in ethanol at reflux conditions. The compounds were confirmed by spectral (IR, 1H & 13C NMR) and elemental analysis. The compounds were screened for their in vitro antioxidant activity against DPPH. We show that compound #4i has potential antioxidant activity. The Molecular docking analysis of the compound with DPPH shows strong hydrogen bonding interactions with several amino acid residues of the protein tyrosine kinase enzyme structure (PDB ID: 2HCK) for effective inhibition.

## Background

Free radicals are a group of unstable substances that are constantly generated for specific metabolic requirement and quenched by an efficient antioxidant network in the body [1-2]. When the generation of these species exceeds the levels of antioxidant mechanism, it leads to oxidative stress, which results in the damage of tissues and biomolecules leading to conditions such as inflammation, carcinogenesis and atherogenesis [3- 4]. Synthesis and evaluation of antimicrobials to protect from free radicals is of interest [5]. Chalcones [6] are a type of open chain flavonoids bearing two aryl rings connected through a three-carbon spacer, the propenone linkage. The reactive α, β unsaturated propenone fragment is not only responsible for the bioactivities but is also useful for the conversion of chalcones to different classes of heterocyclic compounds. There is a difference in the intensity of the bioactivities shown by chalcones due to variation in the nature and type of aryl rings. Chalcones containing heteroaromatic rings in their aryl portion showed excellent biological profiles [7- 8]. Considerable attention has been given to isooxazoline derivatives due to their varied pharmacological activities like anti-microbia [6], anti-inflammatory [7], anti-tubercular [8], antidepressant [9], antioxidant [10], antitumor [11] and DNA Methyltransferase Inhibitors [12-13]. Therefore, it is of interest to document the molecular docking analysis of isoxazoline derivatives with DPPH followed by in vitro validation.

## Materials and Methods:

Chemicals are purchased from chemical company of Aldrich Bangalore. The Suntex melting point instrument was used for to check the melting points of synthesized compounds. The UV spectra of substituted oxazoles have been noted using ELICO BL222 Spectrophotometer. Infrared spectra (Potassium bromide) were recorded in AVATAR spectrophotometer. The synthesized isoxazole compounds NMR spectra are recoded by BURKER spectrometer-500MHz with.

Stage I: Preparation of 3-(4-fluorophenyl)-1-phenylprop-2-en-1-one:

Appropriate mixture of 4-Fluorobenzaldehyde (100mmol) with m- and p-substituted acetophenones (100mmol), solution of aqueous NaOH (200 ml 0.5M) and pure ethyl alcohol were taken in a beaker. The reaction mixture was stirred well vigorously for 30 minutes [12,13] at 30°C. The conversion of aldehydes monitored using TLC, then the mixture to allow for non-disturbed condition for half an hours, then the unreacted reactants are removed with filter paper and washed for using deionized water. The 3-(4-fluorophenyl)-1-phenylprop-2-en-1-ones is recrystallized with ethyl alcohol. The same procedure maintained for the substituted 3-(4-fluorophenyl)-1-phenylprop-2-en-1-ones.

Stage II: Preparation of 5-(4-fluorophenyl)-3-phenylisoxazole:

The synthesized stage I compound 3-(4-fluorophenyl)-1-phenylprop-2-en-1-one (100mmol), hydroxylamine hydrochloride (100mmol) and 0.5 gram sodium hydroxide in 20 mL ethyl alcohol. These contents are condensed for heated to 4 hour [14,15]. The thin layer chromatography technique is followed for the product checked in this reaction. Then, the contents are poured to ice pieces with water. Then the unreacted reactants are removed with filter paper and washed for using deionized water. The 5-(4-fluorophenyl)-3-phenylisoxazoles are recrystallized with ethyl alcohol. The similar procedure maintained for substituted 5-(4-fluorophenyl)-3-phenylisoxazoles. The synthesized 5-(4-fluorophenyl)-3-phenylisoxazoles may conform by physical and spectral data. The above compound physical and spectral data are given in Table 1(see PDF). In the current study the spectral data linearity of synthesized 5-(4-fluorophenyl)-3-phenylisoxazoles have been evaluated the substituent effect tool. Spectral values for the 5-(4-fluorophenyl)-3-phenylisoxazole, UV max(nm), infrared ϑC=N, ϑC=C, ϑC-H, the 1H of C-H, and the 13C C=N, CH, CH-Ph and C-F values are conformed and given Table 2(see PDF).

## Antioxidant activity:

DPPH (2,2-Diphenyl-1-picrylhydrazyl) free radical scavenging assay was evaluated for the antioxidant activity of title compounds [14-15]. Initially, the percentage of inhibition against their corresponding concentrations was assessed from the results obtained for the blank (without test solution) and test (analyte) samples, and compared to the control (standard) sample (Ascorbic acid). The percentages of values for inhibition were determined using the equation

% of inhibition = [(A_Control_ - A_Test_)] / (A_Control_) * 100. 

Where, Acontrol is the control absorbance i.e., L-Ascorbic acid and ATest is the absorbance of synthesized compounds. All the tests were performed in triplicate and their mean and standard deviations were given in values. Each experiment was repeated in three separate assays, and inhibition percentages were observed as concentration dependent dose. Similarly, half of the maximum inhibitory concentration (IC50) values, i.e., the concentration required to decrease the absorbance to 50 percent by both scavenging assays was also determined by plotting the linear curves for the graphs plotted as the percentage of inhibition against the rates.

In the DPPH radical scavenging process 0.1mM of methanolic DPPH solution was prepared, and 1.0mL of this solution was applied to 3.0mL of different concentration methanolic test solutions (50, 100, 150, 200µg/mL). The mixture was vigorously shaken in the dark and incubated for 45min at room temperature. After that the reduction of DPPH free radical scavenging activity was determined by calculating the absorbance spectro-photometrically at 517 nm as a percentage of the inhibition from DPPH discoloration readings. Higher sample absorption suggests greater radical scavenging activity [16].

## Molecular docking studies:

The crystal structure of protein human cyclin-dependent kinase 2 complex (PDB ID: 1HCK) enzyme was obtained from the Protein Data Bank (PDB) and was used in this study [17]. In general, the protein structures are refined for their bond orders, formal charges and missing hydrogen atoms, topologies, incomplete and terminal amide groups. The water molecules beyond 5Å were removed. The possible ionization states were generated in the protein structure and the most stable state was chosen. The hydrogen bonds were assigned and orientations of the retained water molecules were corrected. Finally, a minimization of the protein structure was carried out using OPLS2005 force field to reorient side-chain hydroxyl groups and potential steric clashes. The minimization is restrained to the input protein coordinates by a predefined Root Mean Square Deviation (RMSD) tolerance of 0.3Å.

The ligands structures were generated in the CDX format using Chem Draw ultra version 8.0. These ligands were then converted to the mol2 format and the ligands were prepared by LigPrep module of Maestro in the Schrodinger suite 2013. They were converted from 2D to 3D structures by including stereo chemical, ionization, tautomeric variations, as well as energy minimization and optimized for their geometry, desalted and corrected for their chiralities and missing hydrogen atoms. The bonds orders of these ligands were fixed and the charged groups were neutralized. The ionization and tautomeric states were generated between pH of 6.8 to 7.2 using Epik module. In the LigPrep module, Optimized Potentials minimized the compounds for Liquid Simulations-2005 (OPLS-2005) force field in Impact package of Schrodinger until a RMSD of 1.8Å was achieved. A single low energy ring confirmation per ligand was generated and the optimized ligands were used for docking analysis.

The ligand adenosine-5'-triphosphate was retained in the crystal structure of the prepared protein which was used for the receptor grid construction. The binding box dimensions (within which the centroid of a docked pose is confined) of the protein was set to 17ao x 17ao x 17ao. The accuracy of the docking studies are determined by finding how closely the lowest energy pose of the co-crystallized ligand predicted by the object scoring function, Glide score (G Score), resembles an experimental binding mode as determined by X-ray crystallography. The Glide docking procedure removed the co-crystallized ligand from the binding site of the protein. The hydrogen bonding interactions and the RMSD between the predicted conformation and the observed X-ray crystallographic conformation were used for analyzing the results.

The glide docking of the designed molecules was carried out using the receptor grid and the ligand molecules. The favorable interactions between ligand molecules and the receptor were scored using Glide module of ligand docking program. All the docking calculations were performed using extra precision (XP) mode. The docking process was run in a flexible docking mode, which automatically generates conformations for each input ligand. The ligand poses generated were passed through a series of hierarchal filters that evaluate the ligand's interaction with the receptor. The spatial fit of the ligand to the defined active site, and examines the complementarity of the ligand receptor interactions using grid-based method by the empirical Chem Score function. This algorithm recognizes favorable hydrogen bonding, hydrophobic, metal-ligation interactions, and penalizes steric clashes. Poses that pass these initial screens enter the final stage of the algorithm, which involves evaluation and minimization of grid approximation OPLS non-bonded ligand-receptor interaction energy. Finally, the minimized poses were re-scored using Glide Score scoring function. The XP-Glide score of active compounds were summarized and the fitness scores for each ligand in human cyclin-dependent kinase 2 are compared. When compared with the G-score of standard compound containing ascorbic acid derivative, which is used as antioxidant agent, most of the proposed compounds have good Glide scores [18]. The predicted ADME properties of the proposed compounds were determined by qikprop of Schrodinger software maestro 11.0 version [19].

## Results and Discussion:

The characterization of final compounds was done on the basis of FTIR, mass, 1H NMR and 13C NMR spectral data. In IR spectra, the final compounds (4a-i) showed the presence of -C=N absorptions around 1597 cm-1 and 1601 cm-1 due to isoxazoline ring. Moreover, a sharp absorption band around 1219-1229 cm-1 was observed in all the spectra due to C-O-N stretching. In 1H NMR, the CH protons of isoxazoline ring appeared as singlets around d 6.9. The three protons of methyl group appeared as singlet around d 2-3 and as usual aromatic protons appeared in the range of d7.0-8.0. Moreover, the different carbons present in the synthesized molecules were observed at the expected chemical shifts and integral values in 13C NMR spectra. The physical and spectral data of the compounds are shown in Table 1 & Table 2(see PDF).

**Figure 1:**
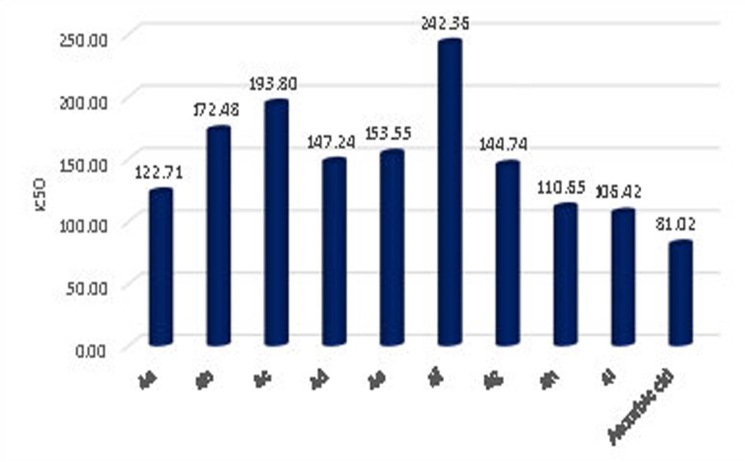
Antioxidant activity of isooxazoline derivative (4a-i) by using DPPH radical method

**Figure 2:**
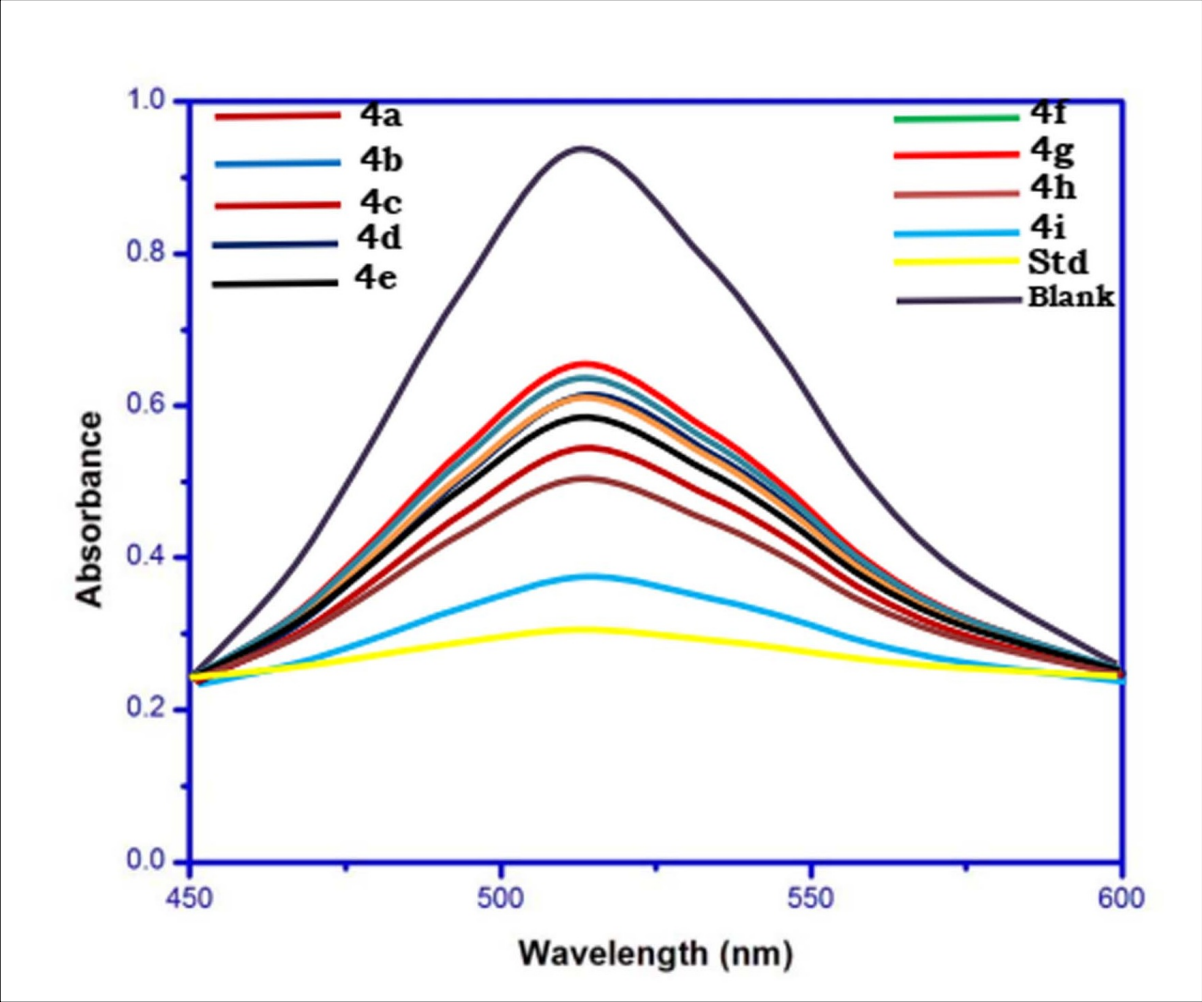
Typical absorption spectra of 50 μg/mL of the DPPH radical alone and in presence of a 100 μg/mL concentration of isoxazoline 4a-i and Ascorbic acid.

The absorbance ranges from 515 - 520 nm for DPPH dissolved in ethanol. The solution with a substance can donate a hydrogen atom and it gives rise to the reduced form by losing its purple color. The reducing ability of the compounds was determined at 100 µg/mL concentrations with DPPH spectro photo-metrically using UV-Vis by considering the decrease in absorbance at 517 nm. Identification of new and potent antioxidants is a major goal for pharmaceutical and medicinal chemistry researchers in order to remove excess of free radicals. In such search we have succeeded by identifying isoxazoline moiety and modifying its structure by introducing some heteroatom containing rings on it to enhance its activity. From Table 3(see PDF), it can be concluded that, as the concentration of the synthesized compounds increases, the absorbance value of DPPH radical decreases. According to previous work the lower absorbance of DPPH radical in the mixture indicates the higher free radical scavenging activity. In addition, the unpaired electron by the whole compound is due to the stability of DPPH and the reduction is measured by the decrease in absorbance. Thus, DPPH shows a strong absorption band at 517 nm due to its odd electron and the absorption vanishes as the electron pairs off. This results to decolourization and stoichiometric with respect to the number of electrons taken up. The RSA value of each compound at different concentration is calculated and expressed as a percentage of the ratio of decrease in absorbance at 517 nm to the absorbance of blank solution (DPPH without samples) at 517 nm (Table 3(see PDF); Figure 1 and Figure 2).

From the obtained DPPH free radical scavenging assay results it is identified that 4a-i are showing IC50 concentrations in the range of 106.42 - 242.36µg/mL, ascorbic acid is showing 81.02 µg/mL. From these results it is identified that the analyzed compounds are following the decreasing order of antioxidant activity as "ascorbic acid > 4i > 4h > 4a> 4g> 4d>4e>4b>4c>4f. These results approve compound 4i as a potential antioxidant with a comparable potency of ascorbic acid reference, which is supported by the obtained percentage of scavenging results at 50, 100, 150 and 200 µg/mL concentrations.

From the results, it can be concluded that RSA in DPPH increases with increase in concentration and it is in correlation with the previous study. The scavenging activity of the compound increases significantly, with increasing concentrations. The nine newly synthesized isoxazoline derivatives are organic compound that are substituted with phenyl ring as electron withdrawing and donating group. As shown in Table 3 (see PDF), compound 4i shows the highest RSA among the compounds as compared to the standard. This is due to the para substitution of mono methyl on the phenyl of isoxazoline ring. Previous study reported that para substitutions of electron donating group are effective, while ortho and meta substitution decreases the antioxidant activity. Ortho and meta substitution causes steric hindrance which decreases the availability of hydrogen radical to combine with DPPH. It was reported that the presence of chloro group in position 3 seems to increase antioxidant activities. However, in this study, compound 4f shows low activity, which is due to the presence of fluorine group on the phenyl ring at position 4, which attached to the isoxazoline ring. He further stated that the position and the type of substituent on the aromatic part of isoxazoline molecule have high influence on the radical scavenging ability.

ADME profile of drug like molecules is very important. The Schrodinger's maestro molecular modeling package Qikprop module was utilized for this work. The Absorption, distribution, metabolism and excretion descriptors of the designed molecules are given in Table 5(see PDF). Blood brain barrier partition coefficient (QPlogBB), estimated IC50 value for HERG K+ channels obstruction (log HERG), estimation of human serum albumin binding (QPlogKhsa), permeation through skin estimation (QPlogKp), apparent Caco 2 cell permeability estimation in nm/sec (QPPCaco) and apparent MDCK cell permeability estimation in nm/sec (QPPMDCK), partition coefficient in octanol and water Log P, solubility in aqueous media log S, Lipinski's rule of five and Percent Human Oral Absorption (%HOA). Results revealed that ADME parameters of each ligand within the bounds of satisfactory range without violating Lipinski's rules. Estimated number of hydrogen bonds that would be accepted by the solute from water molecules in an aqueous solution of the compounds is in the range of 1.5. Predicted octanol/water partition coefficient values of the compounds are in the range of 4.36 - 4.80. The compounds have highest QPlogP value. Number of violations of Lipinski's rule of five is 0. All the compounds have % Human Oral Absorption of 100%. So almost all the properties of the compounds are within the recommended values.

**Figure 3:**
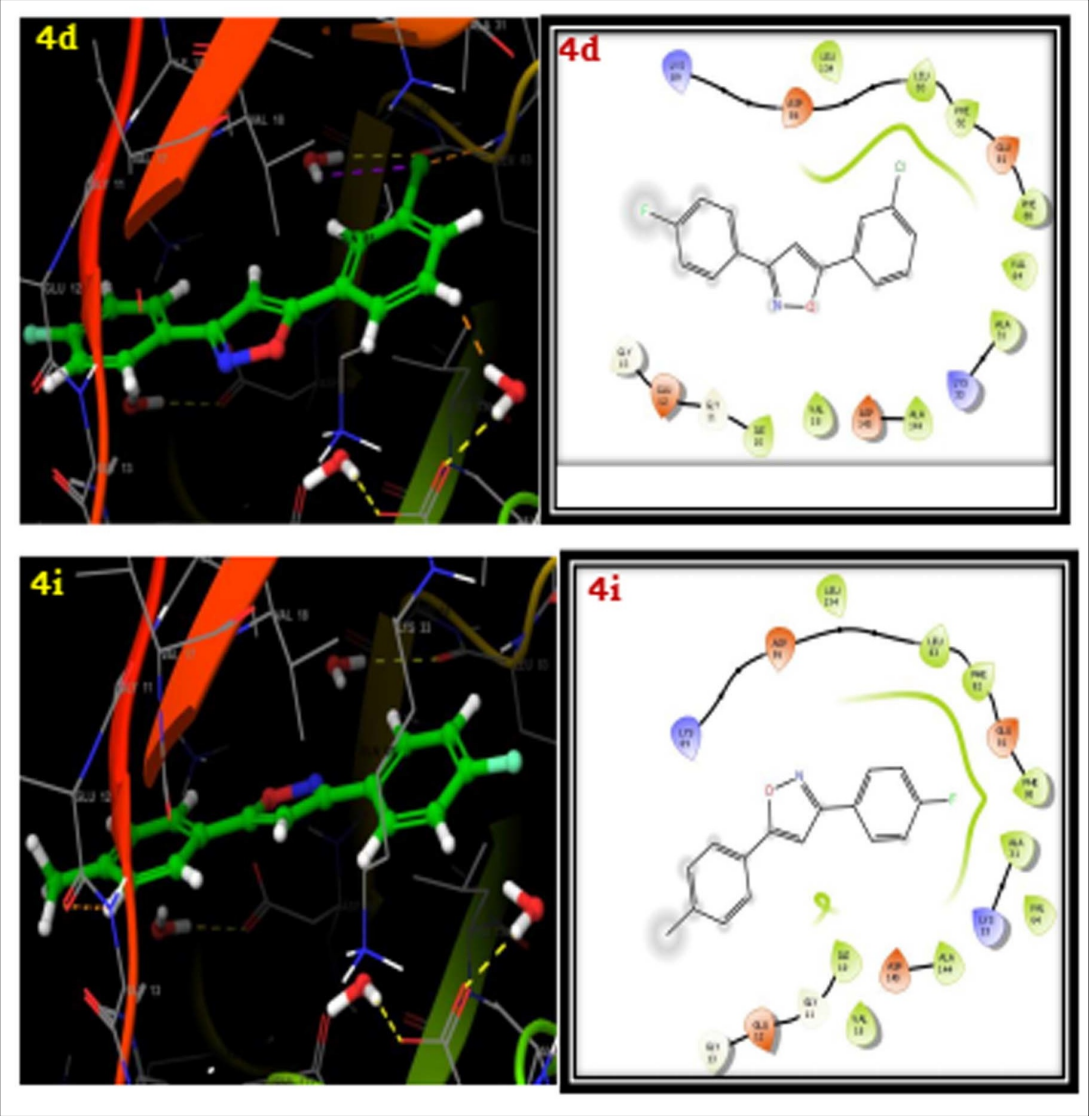
3D docking results of synthesized isooxazoline derivatives (4a-i) with 1HCK

**Figure 4:**
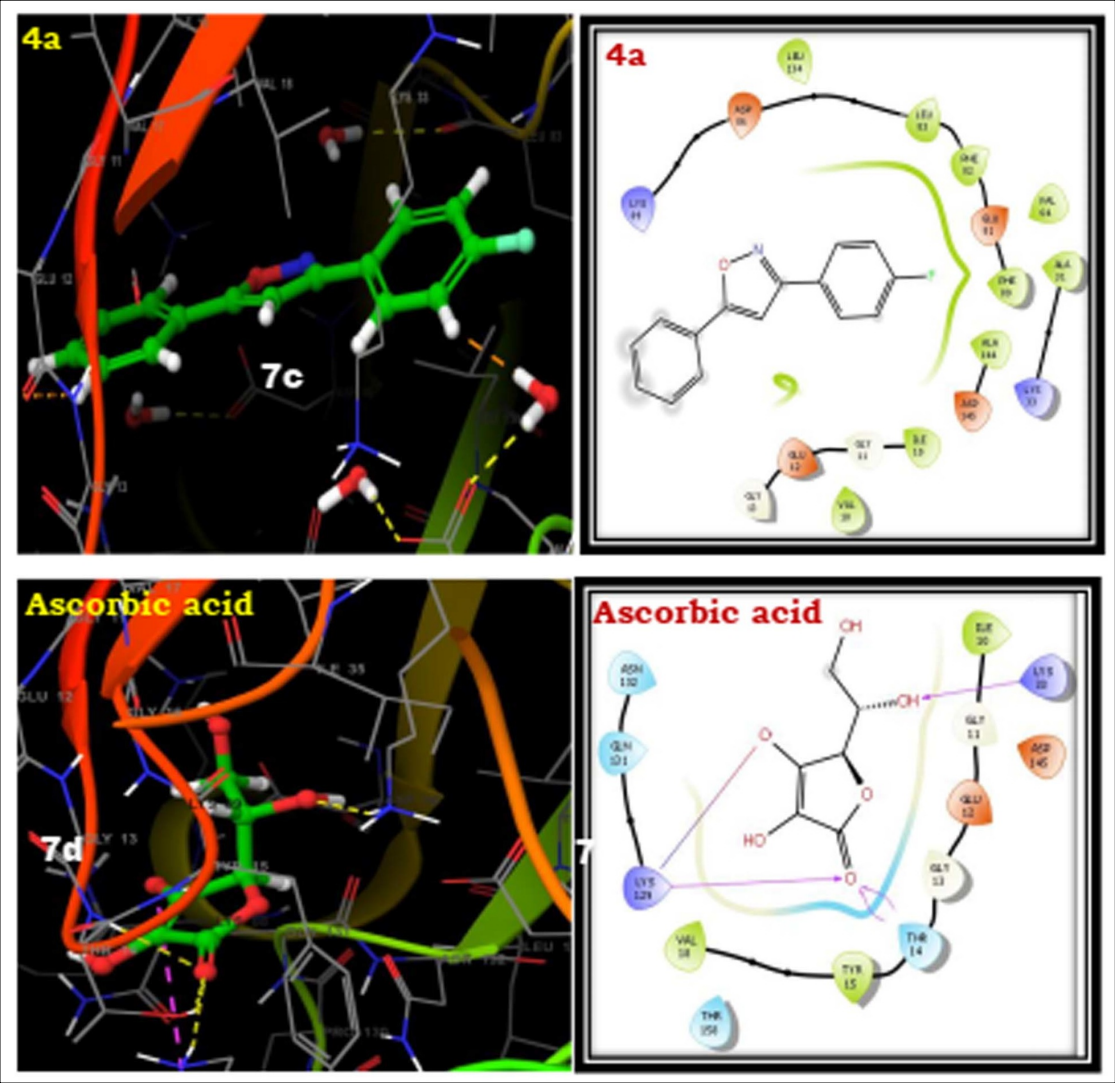
3D docking results of synthesized isooxazoline derivatives (4a-i) with 1HCK

The docking studies of the ligands to protein active sites were completed using the Schrodinger Maestro-11.2 version for determining the binding affinities of the compounds. The designed analogues are docked with the human cyclin-dependent kinase 2 complex (PDB ID: 1HCK). The compounds 4a-i showed good affinity to the receptor when compared with standard ascorbic acid derivative. The compounds 4a, 4d and 4i have more Glide scores due to more lipophilic character. The compounds 4a, 4d and 4i have more active due to the more lipophilic groups such as phenyl, 3-chloro and 4-methyl respectively. The results are summarized in the Table 4. The best affinity modes of the docked compounds (4a, 4d and 4i) with human cyclin-dependent kinase 2 complex (PDB ID: 1HCK) having good Glide score are shown in Figure 3 & Figure 4.

## Conclusion

We show the molecular docking analysis of the compound (#4i) with DPPH having strong hydrogen bonding interactions with amino acid residue of protein tyrosine kinase (2HCK) enzyme for effective inhibition supported by in vitro validation.
